# Non‐recurrent laryngeal nerve and arteria lusoria: Rare and little known association

**DOI:** 10.1002/ccr3.4723

**Published:** 2021-08-25

**Authors:** Azza Mediouni, Hela Sayedi, Houda Chahed, Ghazi Besbes

**Affiliations:** ^1^ University of Tunis El Manar Medical school of Tunis Tunis Tunisia; ^2^ ENT Department Rabta Hospital Tunis Tunisia

**Keywords:** aberrant subclavian artery, CT scan, laryngeal nerve, non‐recurrent laryngeal nerve, thyroidectomy, ultrasonography, vocal palsy

## Abstract

Non‐recurrent Laryngeal nerve is constantly associated with Arteria Lusoria. Knowing this association is the basis of predicting this condition preoperatively. Ultrasonography assessment before thyroid and parathyroid surgery should include identification of brachiocephalic trunk division. Absence of its visualization indicates Arteria Lusoria and then non‐recurrent laryngeal nerve.

## Graphical abstract

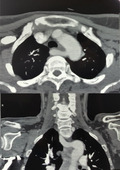

## 1 INTRODUCTION

Non‐recurrent laryngeal nerve (NRLN) is an extremely rare entity constantly associated with an aberrant right subclavian artery also called arteria lusoria. Knowing this association can help to predict a NRLN preoperatively and thus to prevent its injury. We present two patients who had a NRLN, predicted preoperatively by the presence of an arteria lusoria on the CT scan in the first case, discovered during surgery and associated to arteria lusoria on CT scan, practiced postoperatively, in the second one. We will discuss the embryological basis, the radiological features of this rare association and the different methods to predict preoperatively a NRLN, through the cases of two patients in whom NRLN associated with ARSA was proven.

Non‐recurrent laryngeal nerve (NRLN) is a rare entity associated with an aberrant right subclavian artery (ARSA) also called arteria lusoria.^1^ This association is not well known by surgeons or by radiologists.^2^ Being aware of it can help to predict the presence of a NRLN before surgery and thus avoid its injury.

In our department, between January 2016 and December 2018, a total of 472 total thyroidectomies, right lobectomies, and right parathyroidectomies have been done. We do not use intraoperative neuromonitoring (IONM), but dissection of inferior laryngeal nerve is systematic. Four NRLN have been identified. Its frequency is estimated to 0.8%.

## 2 CASE REPORTS

### 2.1 Case 1

**FIGURE 1:**
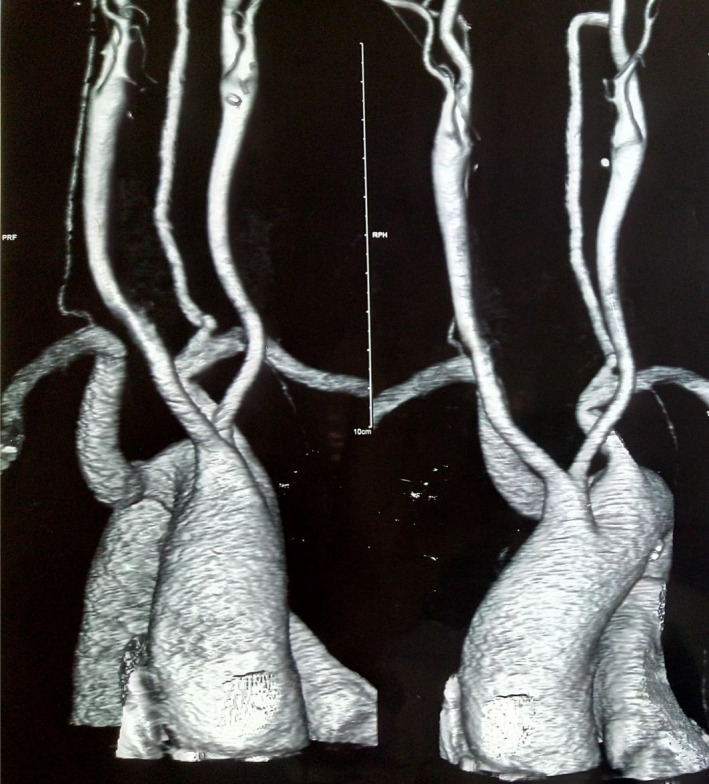
3D reconstruction of contrast‐enhanced CT scan showing an aberrant subclavian artery (lusoria)

**FIGURE 2:**
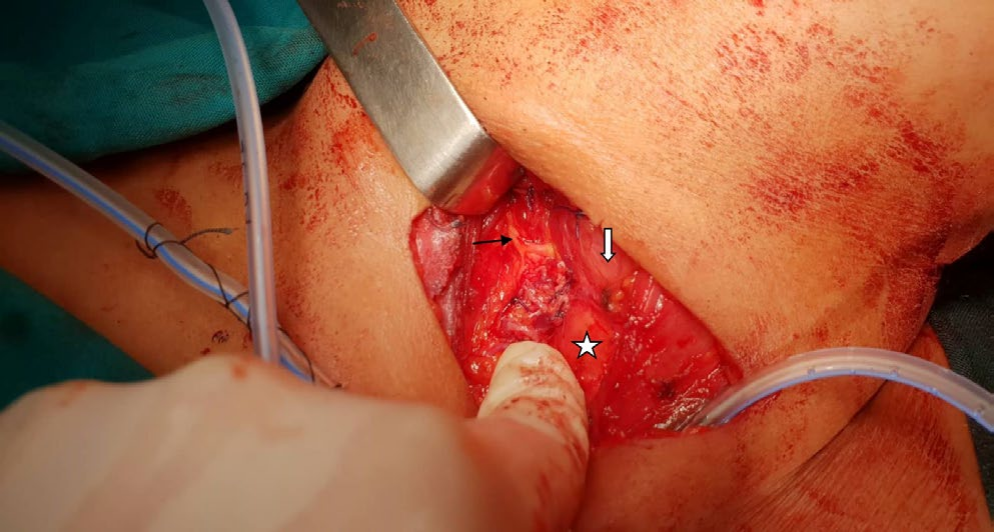
NRLN (black arrow), intraoperative view after thyroidectomy. Linear course at the level of upper thyroid pole entering the larynx (white arrow). Trachea is shown by white star

A 60‐year‐old woman presented with a multinodular goiter. Ultrasonography (US) showed intra‐thoracic extension of goiter. A CT scan has been done and confirmed plunging goiter. CT scan discovered also an ARSA compressing the esophagus (Figure 1). Knowing this, the surgeon was aware of the presence of a NRLN. The patient underwent a total thyroidectomy. A NRLN has been found. It had a linear course at the level of the upper thyroid pole (Figure 2). Identification and dissection were simple; no vocal palsy was noted postoperatively.

### 2.2 Case 2

**FIGURE 3:**
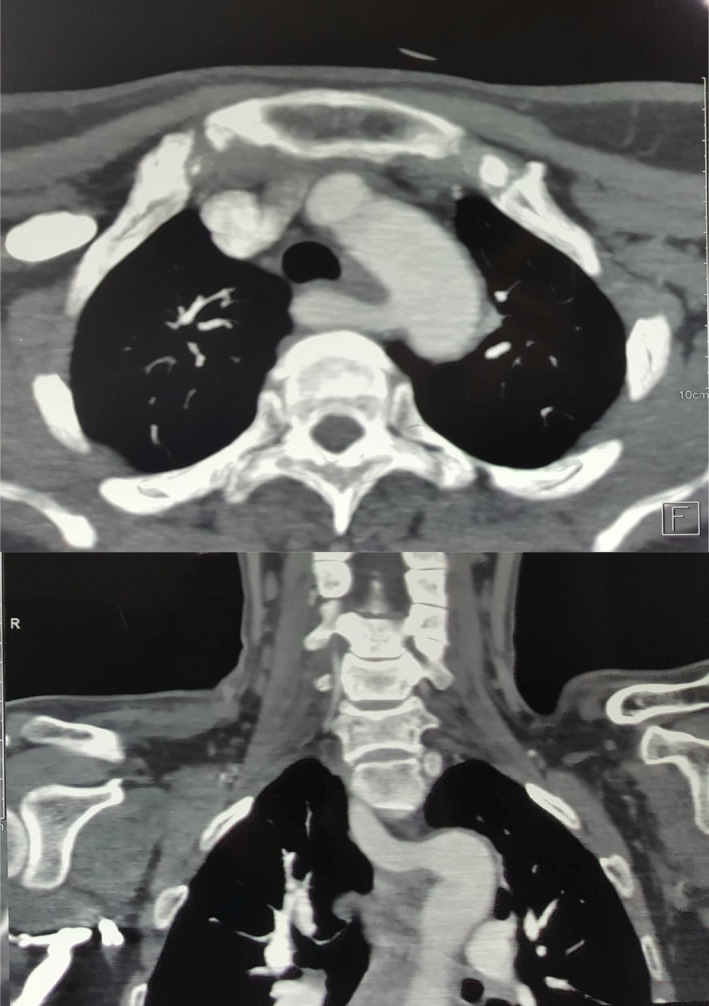
Axial and coronal CT scan, arterial phase, showing an ectopic vessel originating from the aortic arch and crossing the midline between the esophagus and the vertebral column to form the right subclavian artery (arteria lusoria)

A 56‐year‐old woman has been referred for primary hyperparathyroidism. Ultrasonography showed an inferior right parathyroid adenoma measuring 7*18mm. Parathyroid scintigraphy confirmed right inferior parathyroid adenoma. The patient underwent a parathyroidectomy. The right laryngeal nerve has been found non‐recurrent during surgery, after unsuccessful investigation in classic landmarks. It had a linear course transversally at the level of the thyroid isthmus. Surgical outcome was good with no vocal palsy. Since the patient suffered from dysphagia, ARSA was a possible explanation to it. After the patient's consent, CT scan was performed and confirmed an ARSA (Figure 3).

## 3 DISCUSSION

NRLN is a rare anatomic condition. It was first described by Stedman in 1823. Its incidence is reported to range from 0.6 to 1.3% on the right side,^3^ although some reports showed it more frequent when detected by systematic intraoperative neuromonitoring (IONM).^4^ It is exceptionally located on the left side, only in case of dextrocardia or in situs inversus; its incidence is 0.04% in this situation.^3^

This anomaly originates from a malformation of the aortic arch and it is associated with an ARSA.^2^ During the embryological development and as the heart descends, inferior laryngeal nerves assume their recurrent course hooking around the sixth branchial arch. On the left side, the 6^th^ aortic arch remains until birth forming the ductus arteriosus and later ligamentum arteriosum, the left inferior laryngeal nerve keeps its recurrent path in the mediastinum. In the right side, however, the 5^th^ and the distal part of the 6^th^ aortic arches disappear, the right laryngeal nerve ascends to the larynx as high as the 4^th^ aortic arch. The right 4^th^ aortic arch gives birth to the initial segment of the right subclavian artery. An embryological anomaly consisting in the obliteration of the right 4th aortic arch can be seen, the right subclavian artery takes off below the left subclavian artery crossing the midline to irrigate the right arm. Thus, the right laryngeal nerve arises from the vagus in the cervical region passing directly to the larynx without any recurrent path.^2^

The higher incidence of nerve injury, estimated to 12.9% on NRLN compared to 1.8% on recurrent laryngeal nerve,^3^, ^5^ and the absence of reliable clinical signs of a NRLN^6^ exhorted many authors to look for ARSA preoperatively to predict a NRLN.

There are different methods to identify an ARSA.

MRI and CT scan can find it, although it can be missed falsely for technical considerations^6^, ^7^, ^8^ or not mentioned by the radiologist in the final report.^7^ The right subclavian artery can sometimes be oppressed dorsally by the thyroid tumor mimicking an ARSA.^8^ Furthermore, MRI and CT scan are not recommended for all patients who will undergo a thyroid or parathyroid surgery.^9^

Some recent reports suggest the use of ultrasonography (US) as a useful tool to predict a NRLN.^7^, ^9^, ^10^, ^11^, ^12^, ^13^, ^14^ It is a simple, rapid, non‐invasive, reliable, and cost‐effective method,^7^, ^14^ it is also included in the preoperative assessment before thyroid surgery.^7^ Its sensibility and sensitivity varies between 99–100% and 41–100%.^1^

For Devèze and al, it tooks 5mn on ultrasonography of the brachiocephalic trunk, to prove an arteria lusoria on patients with known NRLN. The absence of the brachiocephalic artery and the direct origin of the right common carotid artery from the aorta arch were assessed.^14^ In the controlled trial of Iacobone and al, the surgeon performed preoperatively an ultrasonography of brachiocephalic trunk in one group and none in the control one. The examination aimed to visualize the presence of the division of the brachiocephalic artery into the right common carotid artery and the right subclavian artery (“Y sign”). When the division of the brachiocephalic artery and the subclavian artery was not immediately evident, the course of the right common carotid artery was traced in order to identify its possible origin directly from the aortic arch. The absence of the “Y sign” indicates the presence of a NRLN. Results from this study proved that absence of “Y sign” predicted NRLN with an accuracy of 100% and showed that mean time to identify laryngeal nerve in group with preoperative ultrasonography was shorter.^7^ Frequency of laryngeal nerve palsy was significatively lower in predicted NRLN group (0/5) compared to NRLN discovered preoperatively (3/4).^7^

IONM can also predict the presence of a NRLN by showing negative electromyographic signals from the lower portion (inferior border of the fourth tracheal ring) but positive responses from the upper portion of the vagus nerve (superior border of the thyroid cartilage) after its stimulation.^6^, ^15^

## 4 CONCLUSION

NRLN is a rare anatomic condition constantly associated with ARSA. It is important to be aware of this association since it can help to predict a NRLN preoperatively and avoid its injury. IONM seems to be an effective tool to identify NRLN preoperatively.

## CONFLICT OF INTEREST

The authors declare no conflict of interest.

## AUTHOR CONTRIBUTIONS

Azza Mediouni: Definition of intellectual content, Data analysis, Literature search, Draft Revision, and Manuscript preparation. Hela Sayedi: Data acquisition, Literature search, Data analysis, and Drafting manuscript. Chahed Houda and Ghazi Besbes: Revising critically the draft.

## ETHICAL APPROVAL AND CONSENT TO PARTICIPATE

Since it is a retrospective study, no consent to participate was needed. The patient's files were analyzed respecting anonymity.

## Data Availability

Data sharing not applicable to this article as no datasets were generated or analysed during the current study.
